# Supplementary material for: How to Estimate Optimal Malaria Readiness Indicators at Health-District Level: Findings from the Burkina Faso Service Availability and Readiness Assessment (SARA) Data
Source: Int J Environ Res Public Health. 2020 Jun 1;17(11):3923. doi: 10.3390/ijerph17113923 (PMC7312483; doi:10.3390/ijerph17113923)
Supplement: Supplementary file 1 [file ijerph-17-03923-s001.pdf]

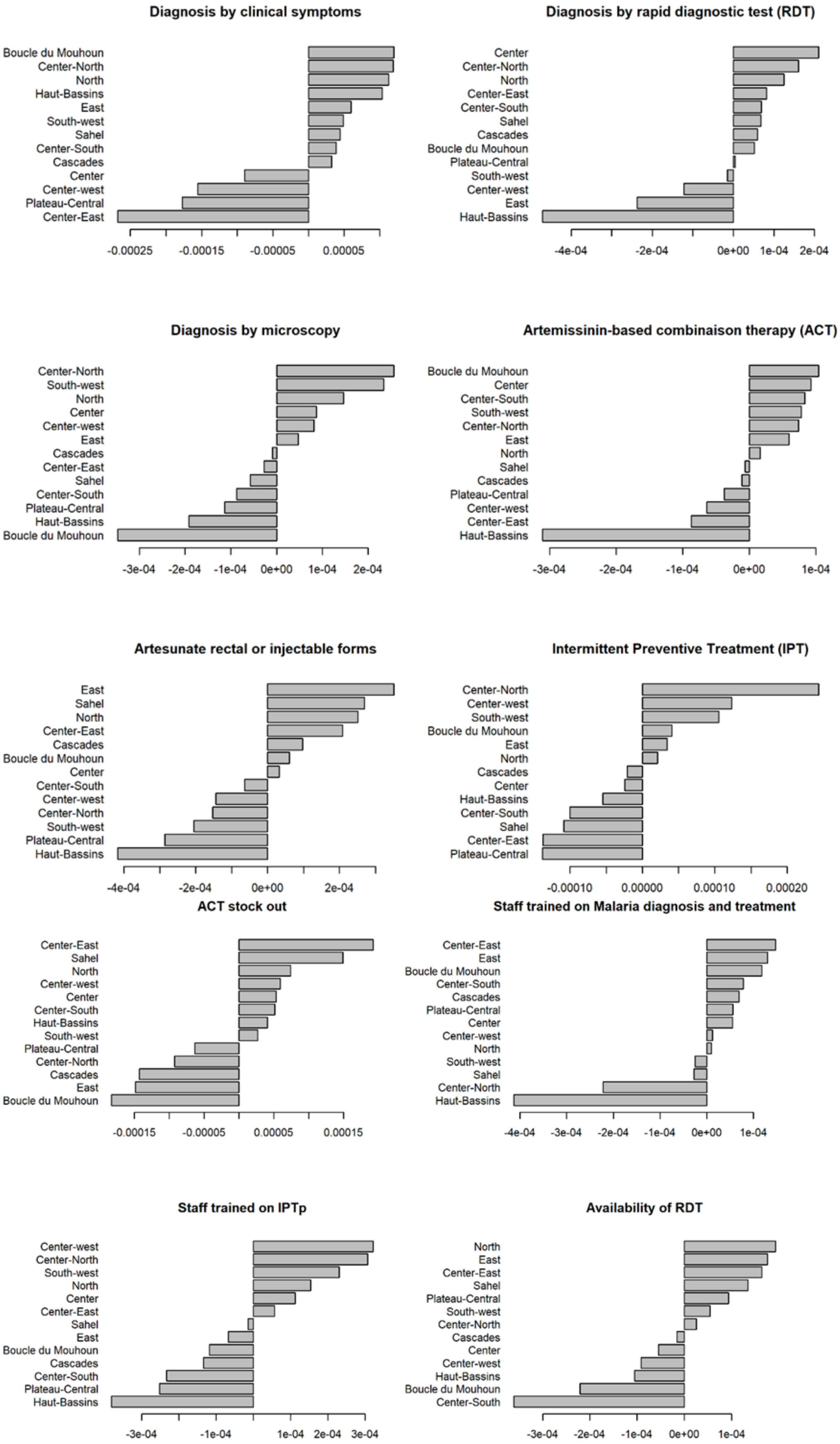

Figure S1. Regional variability for malaria readiness indicators throughout the study area.

Table S1. Geographical distribution of the availability of essential equipment at the health district level: posterior means of fitted values.

Region	Health District	Basic Equipment Availability, % (95% Credible Interval, CrI)						
		Adult weighing scale	Infant weighing scale	Stethoscope	Latex gloves	Thermometer	Tensiometer	Light source
Boucle Du Mouhoun	Boromo	95.8 (93.6–97.5)	82.3 (64.5–93.4)	98.9 (97.8–99.6)	72.6 (48.8–89.9)	99.8 (99.4–100)	94.2 (91.8–96.2)	37.0 (15.8–60.8)
	Dedougou	96.0 (94.1–97.6)	83.5 (67.3–93.7)	98.8 (97.7–99.5)	74.4 (53.6–89.2)	99.7 (99.1–100)	95.3 (93.4–97.0)	53.6 (31.3–75.2)
	Nouna	96.2 (93.7–98.0)	89.9 (78.9–97.4)	98.4 (96.8–99.4)	73.0 (52.4–89.0)	99.4 (98.4–99.9)	95.8 (93.6–97.6)	55.5 (34.6–75.5)
	Solenzo	96.0 (93.9–97.6)	89.6 (77.9–97.3)	98.8 (97.7–99.5)	91.2 (75.7–99.0)	99.7 (99.2–100)	95.0 (92.9–96.8)	62.1 (39.0–82.8)
	Toma	95.8 (93.5–97.5)	88.9 (76.1–97.2)	98.9 (97.9–99.6)	90.6 (75.2–98.5)	99.8 (99.4–100)	94.3 (91.9–96.2)	73.9 (49.4–92.5)
	Tougan	96.0 (93.9–97.7)	83.6 (67.3–93.8)	98.7 (97.5–99.5)	82.4 (63.7–94.4)	99.6 (99–99.9)	95.6 (93.6–97.3)	23.3 (07.2–44.7)
Cascades	Banfora	96.1 (94.2–97.7)	61.3 (41.2–79.3)	98.7 (97.6–99.5)	96.0 (86.9–99.7)	99.7 (99.2–100)	93.4 (90.7–95.6)	38.1 (18.5–59.8)
	Mangodara	95.4 (91.8–97.9)	56.8 (27.7–80.9)	99.1 (97.7–99.8)	88.0 (65.1–98.4)	99.9 (99.6–100)	92.2 (88.2–95.4)	30.0 (06.4–62.1)
	Sindou	96.0 (93.7–97.8)	66.8 (44.9–85.4)	98.9 (97.7–99.6)	96.2 (83.7–99.9)	99.8 (99.3–100)	94.6 (92.0–96.6)	48.8 (24.5–73.5)
Center	Baskuy	95.5 (90.4–98.6)	46.7 (31.1–61.9)	96.2 (91.2–99.0)	87.6 (75.5–95.8)	98.2 (94.6–99.8)	43.8 (33.0–55.0)	84.0 (70.9–93.5)
	Bogodogo	96.1 (94.0–97.8)	62.6 (45.4–77.2)	97.8 (96.3–98.9)	93.7 (85.8–98.4)	99.1 (98.0–99.7)	80.4 (75.5–84.7)	65.3 (48.3–80.3)
	Boulmiougou	96.1 (91.3–98.9)	63.7 (49.7–77.2)	93.9 (86.6–98.3)	85.5 (74.0–93.9)	91.5 (82.0–97.5)	58.3 (46.1–69.9)	75.6 (61.8–87.0)
	Nongr-Massoum	95.7 (91.7–98.4)	64.7 (49.6–79.3)	96.7 (93.1–98.9)	91.6 (81.3–97.8)	98.5 (95.9–99.7)	54.6 (45.2–63.9)	91.4 (80.5–98.0)
	Sig-Nonghin	95.7 (93.0–97.8)	77.0 (61.5–90.0)	98.2 (96.5–99.4)	92.7 (81.1–98.6)	99.6 (98.7–100)	77.3 (70.9–83.1)	80.2 (62.9–92.9)
Center-east	Bittou	95.0 (90.0–98.0)	92.2 (78.3–98.7)	99.1 (97.5–99.9)	97.4 (86.7–100)	99.9 (99.6–100)	91.0 (85.3–95.1)	82.4 (53.8–97.5)
	Garango	95.7 (93.4–97.5)	90.9 (77.3–98.0)	98.9 (97.8–99.6)	96.9 (88.4–99.8)	99.8 (99.4–100)	93.1 (90.2–95.4)	87.1 (67.6–97.9)
	Koupela	95.9 (94.1–97.4)	92.4 (81.5–98.4)	98.8 (97.7–99.5)	98.1 (93.2–99.9)	99.7 (99.2–100)	92.3 (89.6–94.6)	80.2 (59.4–94.2)
	Ouargaye	95.8 (93.7–97.5)	91.3 (78.7–98.0)	98.9 (97.8–99.6)	98.1 (91.9–99.9)	99.8 (99.3–100)	93.7 (91.0–95.8)	55.0 (29.0–78.6)
	Pouytenga	95.4 (92.4–97.7)	92.6 (80.9–98.6)	99.0 (97.7–99.7)	98.1 (92.0–99.9)	99.9 (99.5–100)	92.1 (88.4–95.0)	86.6 (65.9–97.8)
	Tenkodogo	95.5 (92.8–97.6)	92.9 (81.7–98.7)	99.0 (97.8–99.7)	97.7 (92.0–99.8)	99.9 (99.5–100)	93.3 (90.3–95.7)	73.8 (47.4–92.3)
	Zabre	95.2 (90.9–97.9)	92.3 (79.3–98.7)	99.1 (97.6–99.8)	96.3 (84.4–99.8)	99.9 (99.6–100)	91.6 (86.7–95.2)	83.6 (57.5–97.6)
Center-north	Barsalogho	95.3 (91.5–97.8)	85.9 (67.2–97.1)	99.1 (97.7–99.8)	96.7 (86.2–99.9)	99.9 (99.6–100)	92.3 (88.2–95.4)	69.9 (35.8–92.9)
	Boulsa	96.0 (94.0–97.6)	87.1 (73.8–96.2)	98.8 (97.7–99.5)	97.9 (92.8–99.8)	99.7 (99.1–100)	95.3 (93.3–97.0)	83.1 (64.8–95.2)
	Boussouma	94.6 (88.1–98.2)	83.5 (56.9–97.2)	99.2 (97.3–99.9)	96.1 (78.5–99.9)	99.9 (99.7–100)	89.6 (82.0–94.8)	80.1 (36.2–98.2)
	Kaya	96.2 (93.1–98.3)	75.0 (56.7–88.8)	97.8 (95.4–99.3)	96.4 (89.8–99.5)	98.6 (96.7–99.7)	95.6 (92.7–97.6)	92.4 (80.6–98.7)
	Kongoussi	96.0 (94.1–97.5)	88.0 (74.4–97.1)	98.8 (97.7–99.5)	96.2 (88.4–99.6)	99.7 (99.2–100)	94.5 (92.4–96.4)	90.2 (74.8–98.4)
	Tougouri	94.6 (88.2–98.2)	83.6 (57.3–97.2)	99.2 (97.3–99.9)	97.7 (89.6–99.9)	99.9 (99.7–100)	89.6 (82.2–94.8)	80.1 (36.3–98.2)
Center-west	Koudougou	96.3 (91.7–98.9)	83.3 (70.3–93.2)	96.4 (90.7–99.3)	90.4 (79.1–97.5)	95.1 (86.6–99.2)	95.1 (90.8–97.9)	69.3 (52.6–83.8)
	Leo	96.0 (94.0–97.6)	85.4 (71.2–95.2)	98.8 (97.7–99.5)	91.8 (78.9–98.6)	99.7 (99.1–100)	95.3 (93.3–97.0)	59.1 (35.9–80.2)
	Nanoro	95.6 (93.0–97.6)	76.0 (51.9–91.2)	99.0 (97.8–99.7)	75.5 (46.6–93.8)	99.9 (99.4–100)	92.7 (89.6–95.3)	59.9 (32.2–84.1)
	Reo	96.0 (93.9–97.7)	81.6 (65.2–92.6)	98.7 (97.5–99.5)	88.9 (74.8–97.2)	99.6 (99.0–99.9)	95.7 (93.7–97.3)	63.1 (41.2–82.4)
	Sabou	94.7 (88.3–98.2)	81.6 (53.3–96.4)	99.2 (97.3–99.9)	91.9 (66.2–99.6)	99.9 (99.7–100)	89.6 (82.2–94.8)	62.9 (17.7–94.8)

Region	Health District	Basic Equipment Availability, % (95% Credible Interval, CrI)						
		Adult weighing scale	Infant weighing scale	Stethoscope	Latex gloves	Thermometer	Tensiometer	Light source
	Sapouy	95.6 (93.2–97.5)	83.2 (65.1–94.8)	99.0 (97.8–99.7)	89.9 (72.5–98.2)	99.8 (99.4–100)	93.8 (91.1–95.9)	59.2 (31.6–83.6)
	Tenado	94.7 (88.3–98.2)	81.5 (53.2–96.4)	99.2 (97.3–99.9)	89.6 (61.4–99.3)	99.9 (99.7–100)	89.6 (82.3–94.8)	62.9 (17.7–94.8)
Center-south	Kombissiri	95.3 (91.5–97.8)	57.4 (33.7–80.0)	99.1 (97.7–99.8)	91.0 (73.7–98.7)	99.9 (99.6–100)	92.2 (88.1–95.3)	36.0 (12.7–64.0)
	Manga	96.0 (93.9–97.7)	53.3 (32.2–73.1)	98.7 (97.5–99.5)	88.6 (72.5–97.1)	99.6 (99.0–99.9)	95.6 (93.6–97.2)	44.8 (23.1–67.9)
	Po	95.4 (92.2–97.7)	53.3 (29.3–75.9)	99.1 (97.7–99.8)	94.5 (81.3–99.6)	99.9 (99.5–100)	92.7 (89.2–95.5)	28.8 (08.2–55.9)
	Sapone	95.6 (93.2–97.5)	57.7 (34.2–79.9)	99.0 (97.8–99.7)	89.7 (71.7–98.2)	99.8 (99.4–100)	93.8 (91.1–95.9)	28.5 (08.2–55.5)
East	Bogande	95.8 (93.7–97.5)	85.3 (69.8–96.2)	98.9 (97.8–99.6)	98.4 (94.3–99.9)	99.8 (99.3–100)	93.6 (91.2–95.7)	48.7 (22.9–73.9)
	Diapaga	96.0 (94.0–97.6)	80.9 (64.3–93.0)	98.6 (97.5–99.4)	98.2 (91.6–100)	99.6 (98.9–99.9)	94.3 (91.7–96.3)	87.1 (69.5–97.6)
	Fada N'gourma	96.0 (94.1–97.5)	71.3 (50.3–86.7)	98.7 (97.7–99.4)	98.5 (94.4–99.9)	99.7 (99.1–99.9)	94.0 (91.6–95.9)	66.3 (44.5–84.8)
	Gayeri	95.2 (91.4–97.8)	84.1 (66.2–96.1)	99.1 (97.7–99.8)	98.6 (93.2–100)	99.9 (99.6–100)	92.2 (88.1–95.4)	81.3 (55.3–96.8)
	Mani	95.3 (91.5–97.8)	83.7 (65.0–96.0)	99.1 (97.7–99.8)	98.1 (91.9–99.9)	99.9 (99.6–100)	92.2 (88.1–95.3)	62.1 (29.6–88.1)
	Pama	95.0 (90.0–98.0)	77.9 (55.8–92.6)	99.2 (97.5–99.9)	98.6 (92.3–100)	99.9 (99.6–100)	91.0 (85.3–95.1)	75.8 (48.8–94.2)
Hauts-Bassins	Dafra	95.6 (92.1–98.1)	54.2 (34.2–72.2)	98.3 (96.2–99.5)	85.4 (68.7–95.9)	99.7 (98.8–100)	74.2 (65.2–82.0)	77.4 (59.1–91.2)
	Dande	96.0 (94.0–97.6)	82.4 (66.6–94.1)	98.8 (97.7–99.5)	85.4 (66.6–96.4)	99.7 (99.2–100)	95.0 (92.9–96.7)	57.7 (33.7–79.6)
	Do	95.9 (93.2–98.0)	65.4 (48.8–80.7)	98.1 (96.2–99.3)	94.7 (86.1–99.1)	99.5 (98.5–99.9)	75.4 (68.4–81.5)	80.4 (64.4–92.4)
	Hounde	95.8 (93.6–97.6)	75.1 (54.4–89.5)	98.9 (97.8–99.6)	80.7 (59.5–94.3)	99.8 (99.4–100)	94.2 (91.9–96.2)	58.8 (33.3–81.7)
	Karangasso–Vigue	95.2 (91.1–98.0)	80.5 (59.7–94.8)	99.1 (97.6–99.8)	90.8 (72.4–98.9)	99.9 (99.6–100)	91.6 (87.0–95.1)	65.1 (31.5–90.9)
	Lena	95.4 (91.7–97.9)	73.6 (48.0–90.2)	99.1 (97.7–99.8)	90.1 (71.7–98.6)	99.9 (99.6–100)	92.2 (88.3–95.3)	77.0 (47.9–95.8)
	N'dorola	94.8 (88.4–98.3)	77.9 (50.2–94.7)	99.2 (97.2–99.9)	92.5 (63.3–99.8)	99.9 (99.7–100)	89.5 (82.0–94.8)	65.1 (20.8–94.9)
	Orodara	96.2 (93.7–98.1)	84.3 (69.9–94.9)	98.4 (96.9–99.4)	93.5 (81.3–99.1)	99.4 (98.5–99.9)	96.2 (93.9–97.8)	57.9 (36.7–77.5)
North	Gourcy	95.8 (93.6–97.5)	54.8 (34.2–76.3)	98.9 (97.8–99.6)	80.4 (57.2–94.7)	99.8 (99.3–100)	94.6 (92.4–96.5)	25.1 (06.4–50.3)
	Ouahigouya	96.1 (94.0–97.7)	49.6 (32.6–67.2)	98.5 (97.3–99.4)	94.7 (85.4–99.3)	99.5 (98.8–99.9)	94.7 (92.3–96.6)	75.8 (58.1–89.8)
	Seguenega	95.8 (93.7–97.5)	45.6 (24.2–66.7)	98.9 (97.8–99.6)	94.8 (83.9–99.5)	99.8 (99.3–100)	94.6 (92.4–96.5)	82.8 (61.1–96.6)
	Thiou	94.6 (88.1–98.2)	51.5 (20.5–84.2)	99.2 (97.3–99.9)	92.9 (64.0–99.8)	99.9 (99.7–100)	89.6 (82.0–94.9)	64.7 (19.3–95.1)
	Titao	95.6 (92.9–97.6)	54.1 (32.2–77.4)	99 (97.8–99.7.0)	95.9 (86.1–99.7)	99.9 (99.4–100)	92.7 (89.6–95.3)	63.5 (37.0–85.9)
	Yako	96.1 (93.6–97.9)	37.9 (19.3–56.8)	98.4 (96.9–99.4)	89.2 (77.2–96.5)	99.4 (98.4–99.9)	95.8 (93.6–97.5)	68.4 (48.4–85.4)
Plateau Central	Bousse	95.7 (93.4–97.5)	85.8 (67.5–96.2)	98.9 (97.8–99.6)	89.8 (73.6–97.9)	99.8 (99.4–100)	93.1 (90.4–95.4)	86.7 (67.0–97.8)
	Ziniare	96.1 (93.4–98.1)	91.9 (81.2–98.2)	98.4 (96.7–99.4)	93.0 (82.5–98.5)	99.3 (98.2–99.9)	96.4 (94.3–98.0)	69.8 (49.2–86.8)
	Zorgho	96.2 (92.8–98.4)	90.4 (79.0–97.4)	97.9 (95.4–99.4)	96.1 (89.4–99.4)	98.7 (96.7–99.8)	96.5 (94.1–98.3)	77.1 (59.0–90.9)
Sahel	Djibo	96.1 (93.9–97.8)	84.1 (67.2–95.0)	98.4 (97.1–99.3)	92.8 (80.2–98.7)	99.4 (98.5–99.9)	95.0 (92.6–97.0)	85.0 (67.3–96.3)
	Dori	95.4 (92.1–97.7)	89.1 (73.7–97.9)	99.1 (97.8–99.8)	97.6 (90.2–99.9)	99.9 (99.5–100)	92.8 (89.3–95.6)	62.2 (32.1–86.8)
	Gorom-Gorom	95.5 (92.5–97.7)	89.4 (74.6–97.9)	99.0 (97.8–99.7)	96.2 (82.3–99.9)	99.9 (99.5–100)	93.3 (90.0–95.9)	72.0 (44.9–91.7)
	Sebba	95.3 (91.5–97.8)	88.8 (72.5–97.9)	99.1 (97.7–99.8)	98.2 (91.8–100)	99.9 (99.6–100)	92.2 (88.0–95.4)	82.9 (57.3–97.3)
South west	Batie	95.3 (90.9–98.1)	74.5 (50.0–93.4)	99.1 (97.5–99.8)	84.8 (52.9–99.1)	99.9 (99.6–100)	91.7 (86.6–95.4)	78.8 (49.5–96.6)

Region	**Health District**	**Basic Equipment Availability, % (95% Credible Interval, CrI)**						
		Adult weighing scale	Infant weighing scale	Stethoscope	Latex gloves	Thermometer	Tensiometer	Light source
	Dano	96.1 (94.2–97.6)	63.7 (41.9–81.9)	98.8 (97.7–99.5)	80.3 (59.9–94.0)	99.7 (99.1–100)	94.5 (92.3–96.4)	59.4 (35.1–81.2)
	Diebougou	95.6 (92.9–97.7)	73.7 (52.0–91.4)	99 (97.8–99.7.0)	88.2 (70.1–98.0)	99.9 (99.5–100)	93.3 (90.3–95.8)	76.1 (50.4–94.0)
	Gaoua	96.2 (94.1–97.9)	61.1 (39.8–79.2)	98.6 (97.3–99.4)	54.2 (30.5–76.4)	99.6 (98.9–99.9)	95.2 (92.9–97.0)	60.4 (38.0–80.4)
	Kampti	94.8 (88.4–98.3)	69.2 (37.0–92.7)	99.2 (97.2–99.9)	83.8 (42.8–99.1)	99.9 (99.7–100)	89.6 (82.1–94.8)	68.3 (22.7–96.1)

Table S2. Geographical distribution of malaria readiness at the health district level: posterior means of fitted values.

Region	Health District	Diagnostic			Anti-Malaria Drugs			
		Symptom	RDT	Microscopy	Oral ACT	Rectal or injectable	ITP-SP	ACT Out of Stock
**Boucle Du Mouhoun**	Boromo	98.9 (97.8–99.7)	94.9 (92.1–97.1)	13.6 (09.6–18.2)	97.8 (94.2–99.7)	27.8 (10.6–49.2)	94.3 (91.3–96.6)	04.6 (02.6–07.2)
	Dedougou	98.4 (97.1–99.4)	95.3 (92.7–97.2)	10.7 (07.4–14.5)	98.6 (96.2–99.8)	44.3 (24.0–65.8)	95.6 (93.3–97.6)	05.0 (03.0–07.5)
	Nouna	97.5 (95.4–98.9)	95.2 (92.3–97.5)	09.2 (05.9–13.2)	99.0 (96.8–99.8)	59.5 (39.2–78.7)	96.2 (93.7–98.1)	06.1 (03.4–09.5)
	Solenzo	98.6 (97.4–99.5)	95.3 (92.7–97.4)	11.7 (08.0–15.9)	98.4 (95.6–99.8)	56.9 (34.9–78.1)	95.3 (92.7–97.4)	04.8 (02.8–07.3)
	Toma	99.0 (97.8–99.7)	95.1 (92.5–97.3)	13.7 (09.7–18.3)	97.7 (94.1–99.7)	31.2 (12.1–54.4)	94.2 (91.2–96.6)	04.5 (02.5–07.0)
	Tougan	98.2 (96.7–99.2)	95.4 (92.8–97.4)	10.1 (06.9–14.0)	98.8 (96.6–99.8)	35.0 (16.5–56.2)	95.9 (93.4–97.7)	05.2 (03.1–08.0)
Cascades	Banfora	98.7 (97.5–99.5)	94.2 (91.0–96.6)	14.1 (09.9–18.9)	96.5 (90.7–99.2)	18.2 (05.8–35.4)	93.4 (90.0–96.0)	05.0 (02.9–07.6)
	Mangodara	99.4 (98.3–99.9)	94.5 (90.6–97.3)	18.5 (12.3–25.8)	95.3 (87.8–99.4)	51.9 (23.5–80.6)	92.0 (87.1–95.7)	03.9 (01.8–07.1)
	Sindou	98.8 (97.6–99.6)	95.1 (92.1–97.4)	12.6 (08.4–17.5)	98.0 (94.7–99.7)	62.9 (39.1–84.3)	94.8 (91.8–97.2)	04.6 (02.5–07.3)
Center	Baskuy	86.2 (72.5–95.6)	50.2 (33.4–67.0)	56.8 (39.9–73.1)	39.2 (23.3–56.2)	20.1 (08.3–35.2)	59.3 (42.3–75.2)	02.4 (00.0–14.9)
	Bogodogo	97.9 (96.0–99.2)	85.2 (79.2–89.8)	28.0 (21.6–35.1)	68.0 (49.4–82.5)	17.1 (07.1–30.5)	78.4 (68.9–84.4)	06.6 (03.8–10.0)
	Boulmiougou	94.4 (86.4–98.8)	68.0 (54.9–79.7)	42.4 (30.1–55.2)	57.2 (41.1–72.7)	12.6 (04.9–23.5)	56.7 (43.3–69.7)	12.4 (05.1–22.6)
	Nongr-Massoum	97.4 (94.0–99.3)	69.7 (59.1–79.8)	52.3 (41.9–63.0)	31.2 (17.7–46.4)	12.8 (04.4–25.2)	55.3 (44.2–66.9)	04.8 (01.6–09.4)
	Sig-Nonghin	98.9 (97.3–99.7)	85.0 (77.7–90.6)	35.0 (26.7–44.2)	70.5 (54.1–85.4)	26.3 (11.8–44.6)	75.4 (66.2–83.1)	05.5 (02.8–09.4)
Center-east	Bittou	99.5 (98.3–99.9)	94.3 (89.7–97.5)	21.5 (13.6–31.1)	93.4 (82.6–99.3)	59.6 (28.7–87.6)	90.4 (83.6–95.3)	03.7 (01.5–07.4)
	Garango	99.0 (97.8–99.7)	94.4 (91.3–96.8)	15.6 (11.1–20.8)	95.7 (88.4–99.1)	45.6 (22.4–70.4)	92.9 (89–95.7.0)	04.4 (02.4–07.0)
	Koupela	98.8 (97.6–99.5)	93.7 (90.5–96.1)	16.2 (11.9–21.2)	96.5 (91.4–99.4)	31.7 (13.5–53.7)	92.3 (88.8–95.2)	05.2 (03.2–08.0)
	Ouargaye	98.8 (97.5–99.6)	94.7 (91.9–97.1)	14.4 (10.2–19.4)	96.3 (90.0–99.2)	58.0 (34.7–80.2)	93.8 (90.6–96.4)	04.7 (02.7–07.4)
	Pouytenga	99.2 (98.1–99.8)	94.3 (90.8–97.0)	18.1 (12.5–24.6)	94.2 (85.1–98.8)	28.3 (09.3–52.8)	91.8 (87.2–95.3)	04.1 (02.0–06.9)
	Tenkodogo	99.1 (98.0–99.8)	94.9 (91.8–97.3)	15.8 (10.9–21.3)	96.8 (91.9–99.6)	56.8 (31.3–81.1)	93.3 (89.7–96.2)	04.2 (02.2–07.0)
	Zabre	99.4 (98.2–99.9)	94.5 (90.3–97.4)	20.2 (13.3–28.7)	94.6 (85.9–99.3)	36.4 (12.2–65.6)	91.0 (85.1–95.3)	03.8 (01.7–07.3)
Center-north	Barsalogho	99.4 (98.2–99.9)	94.7 (90.9–97.4)	18.7 (12.5–26.2)	95.4 (88.1–99.4)	36.0 (12.0–64.9)	91.9 (87.1–95.8)	04.0 (01.8–07.2)
	Boulsa	98.4 (97.0–99.4)	95.4 (93.0–97.4)	11.3 (07.9–15.5)	98.6 (96.2–99.8)	16.8 (04.6–34.3)	95.6 (93.2–97.6)	04.9 (02.9–07.5)
	Boussouma	99.6 (98.5–100)	94.0 (88.2–97.6)	24.5 (14.7–36.6)	90.2 (72.7–99.1)	41.1 (09.7–79.6)	88.5 (79.4–94.9)	03.5 (01.2–07.5)
	Kaya	96.1 (92.6–98.4)	94.4 (90.5–97.2)	09.0 (05.5–13.6)	98.9 (96.5–99.8)	46.5 (28.4–65.3)	96.0 (93.0–98.2)	07.4 (03.9–12.1)
	Kongoussi	98.6 (97.3–99.4)	95.0 (92.4–97.1)	12.6 (09.1–17.0)	98.1 (95.0–99.7)	46.3 (25.3–68.3)	94.8 (92.1–097)	05.0 (03.0–07.5)
	Tougouri	99.6 (98.5–100)	94.0 (88.3–97.6)	24.6 (14.8–36.5)	90.2 (72.7–99.1)	41.5 (10.0–79.6)	88.5 (79.4–94.8)	03.5 (01.2–07.6)
Center-west	Koudougou	92.2 (83.1–97.7)	92.6 (85.9–96.9)	08.8 (04.6–14.7)	97.6 (91.3–99.7)	19.5 (08.4–33.8)	95.6 (91.0–98.3)	09.6 (04.2–17.6)
	Leo	98.4 (97.0–99.3)	95.4 (92.9–97.4)	10.9 (07.6–14.9)	98.6 (96.1–99.8)	46.6 (25.5–68.6)	95.6 (93.2–97.6)	05.2 (03.1–07.9)
	Nanoro	99.1 (98.0–99.8)	94.3 (90.9–96.8)	16.8 (11.8–22.6)	96.3 (90.7–99.4)	27.7 (09.0–52.0)	92.6 (88.8–95.8)	04.3 (02.3–07.0)
	Reo	98.1 (96.6–99.2)	95.5 (92.9–97.5)	10.1 (06.9–13.9)	98.8 (96.6–99.8)	42.3 (22.8–63.3)	96.0 (93.7–97.8)	05.2 (03.1–08.0)
	Sabou	99.6 (98.5–100)	93.9 (88.1–97.6)	24.4 (14.7–36.3)	90.2 (72.7–99.1)	40.6 (29.6–79.0)	88.6 (79.5–94.8)	03.5 (01.2–07.5)

Region	Health District	Diagnostic			Anti-Malaria Drugs			
		Symptom	RDT	Microscopy	Oral ACT	Rectal or injectable	ITP-SP	ACT Out of Stock
	Sapouy	99.1 (97.9–99.7)	95.0 (92.2–97.3)	14.8 (10.5–20.0)	97.3 (93.0–99.6)	42.1 (18.9–67.7)	93.8 (90.6–96.5)	04.4 (02.4–07.0)
	Tenado	99.6 (98.5–100)	93.9 (88.2–97.6)	24.4 (14.8–36.2)	90.1 (72.6–99.1)	40.3 (09.5–78.8)	88.5 (79.5–94.8)	03.5 (01.2–07.6)
Center-south	Kombissiri	99.3 (98.2–99.9)	94.6 (91.0–97.4)	18.5 (12.4–25.6)	95.5 (88.7–99.4)	28.7 (09.4–53.5)	91.8 (86.8–95.5)	03.9 (01.8–07.0)
	Manga	98.2 (96.6–99.2)	95.5 (93.0–97.5)	10.1 (06.9–14.0)	98.8 (96.6–99.8)	44.8 (24.4–66.3)	95.8 (93.4–97.7)	05.3 (03.1–08.1)
	Po	99.3 (98.1–99.8)	94.8 (91.4–97.3)	17.2 (11.7–23.6)	96.2 (90.5–99.5)	42.2 (18.9–68.0)	92.4 (88.1–95.8)	04.1 (02.0–07.1)
	Sapone	99.1 (97.9–99.7)	94.9 (91.9–97.1)	14.8 (10.5–20.0)	97.3 (93.0–99.6)	41.9 (18.8–67.6)	93.8 (90.6–96.5)	04.4 (02.4–07.2)
East	Bogande	98.9 (97.7–99.6)	94.6 (91.7–96.8)	14.7 (10.6–19.6)	97.4 (93.2–99.6)	55.0 (31.3–78.2)	93.8 (90.7–96.4)	04.7 (02.7–07.3)
	Diapaga	98.3 (96.8–99.2)	94.3 (90.9–96.7)	12.4 (08.6–17.1)	98.1 (94.7–99.7)	66.8 (44.4–86.4)	94.3 (91.0–96.7)	05.6 (03.3–08.5)
	Fada N'gourma	98.5 (97.2–99.4)	94.4 (91.5–96.7)	13.1 (09.3–17.4)	97.8 (94.3–99.6)	42.0 (22.5–63.1)	94.2 (91.3–96.6)	05.5 (03.4–08.2)
	Gayeri	99.3 (98.2–99.9)	94.6 (90.8–97.4)	18.6 (12.4–26.1)	95.4 (88.2–99.4)	58.2 (30.3–84.2)	91.9 (87.0–95.7)	04.0 (01.8–07.2)
	Mani	99.3 (98.2–99.9)	94.5 (90.6–97.2)	18.7 (12.5–26.1)	95.4 (88.1–99.4)	28.1 (07.3–56.1)	91.7 (86.7–95.5)	03.9 (01.8–07.1)
	Pama	99.5 (98.4–99.9)	94.3 (89.7–97.5)	21.3 (13.4–30.8)	91.7 (79.4–98.5)	46.5 (20.9–73.6)	90.4 (83.7–95.4)	03.8 (01.5–07.5)
Hauts-Bassins	Dafra	99.2 (97.7–99.8)	83.3 (73.1–90.6)	38.5 (28.2–49.7)	51.2 (32.2–69.0)	12.4 (03.4–26.4)	71.0 (59.1–81.2)	05.9 (02.6–11.0)
	Dande	98.6 (97.4–99.5)	95.2 (92.6–97.3)	11.7 (08.1–15.8)	97.6 (93.0–99.5)	32.9 (14.1–55.0)	95.3 (92.7–97.3)	04.8 (02.8–07.4)
	Do	98.9 (97.3–99.7)	83.7 (75.5–90.0)	35.0 (25.9–44.5)	50.3 (31.2–67.3)	25.2 (11.7–42.1)	71.8 (61.2–80.5)	06.9 (03.2–12.3)
	Hounde	99.0 (97.8–99.7)	94.9 (92.0–97.0)	13.7 (09.7–18.3)	97.7 (94.1–99.7)	42.5 (20.5–66.6)	94.4 (91.5–96.8)	04.6 (02.6–07.2)
	Karangasso–Vigue	99.4 (98.4–99.9)	94.2 (89.8–97.2)	19.9 (13.0–28.0)	94.3 (85.2–99.3)	28.8 (07.1–58.5)	91.2 (85.7–95.5)	03.8 (01.6–07.1)
	Lena	99.4 (98.3–99.9)	94.5 (90.7–97.2)	18.2 (12.1–25.3)	95.3 (87.9–99.4)	34.7 (11.3–63.7)	92.0 (87.2–95.7)	03.9 (01.8–07.1)
	N'dorola	99.6 (98.6–100)	93.8 (87.9–97.5)	24.2 (14.4–36.2)	89.9 (71.9–99.1)	39.3 (08.7–78.4)	88.5 (79.4–94.8)	03.5 (01.2–07.6)
	Orodara	97.6 (95.5–99.0)	95.5 (92.4–97.7)	08.6 (05.3–12.7)	99.1 (97.2–99.9)	18.6 (06.0–35.9)	96.6 (94.1–98.3)	05.7 (03.1–09.2)
North	Gourcy	98.8 (97.6–99.6)	95.3 (92.7–97.4)	12.7 (09.0–17.2)	98.1 (95.0–99.7)	58.1 (34.8–80.1)	94.8 (92.1–97.1)	04.6 (02.7–07.2)
	Ouahigouya	98.0 (96.5–99.1)	94.8 (92.0–97.1)	11.5 (08.0–15.9)	98.4 (95.7–99.7)	50.6 (32.3–69.1)	94.9 (91.9–97.1)	05.5 (03.3–08.3)
	Seguenega	98.8 (97.5–99.6)	95.3 (92.7–97.3)	12.8 (09.0–17.2)	98.1 (94.9–99.7)	49.5 (26.4–73.2)	94.8 (92.2–97.1)	04.7 (02.8–07.4)
	Thiou	99.6 (98.5–100)	94.0 (88.2–97.7)	24.5 (14.6–36.7)	90.2 (72.5–99.1)	41.4 (09.8–79.9)	88.5 (79.2–94.9)	03.5 (01.2–07.6)
	Titao	99.1 (98.0–99.8)	94.5 (91.4–97.1)	16.8 (11.8–22.7)	96.3 (90.8–99.4)	32.6 (12.6–56.8)	92.6 (88.8–95.8)	04.4 (02.3–07.3)
	Yako	97.4 (95.4–98.9)	95.0 (91.9–97.3)	09.5 (06.2–13.6)	98.5 (95.0–99.7)	45.0 (26.2–64.7)	96.1 (93.6–98.0)	06.0 (03.4–09.3)
Plateau Central	Bousse	99.0 (97.9–99.7)	94.6 (91.7–97.0)	15.6 (11.2–20.7)	95.8 (88.7–99.1)	19.6 (05.2–40.3)	92.9 (89.2–95.7)	04.5 (02.5–07.1)
	Ziniare	97.1 (94.4–98.8)	95.6 (92.6–97.7)	08.1 (05.1–12.0)	99.3 (97.6–99.9)	26.7 (11.3–45.7)	96.8 (94.5–98.5)	06.1 (03.3–09.9)
	Zorgho	95.6 (91.3–98.4)	95.3 (91.7–97.8)	07.4 (04.2–11.6)	99.3 (97.7–99.9)	41.7 (23.9–60.9)	96.9 (94.2–98.7)	06.8 (03.3–11.5)
Sahel	Djibo	97.7 (96.0–99.0)	94.7 (91.6–97.0)	10.7 (07.2–15.0)	97.9 (93.4–99.6)	28.1 (11.8–48.1)	95.2 (92.1–97.4)	06.3 (03.8–09.8)
	Dori	99.3 (98.1–99.8)	94.8 (91.4–97.4)	17.1 (11.5–23.5)	96.2 (90.3–99.5)	53.3 (27.2–79.2)	92.6 (88.4–96.0)	04.2 (02.1–07.3)
	Gorom-Gorom	99.2 (98.0–99.8)	94.9 (91.7–97.4)	16.0 (10.8–22.3)	96.8 (91.8–99.6)	63.6 (37.6–86.5)	93.2 (89.4–96.3)	04.3 (02.2–07.3)
	Sebba	99.4 (98.2–99.9)	94.6 (90.9–97.4)	18.6 (12.4–26.0)	95.4 (88.3–99.4)	57.8 (30.0–83.9)	91.7 (86.6–95.5)	04.0 (01.8–07.2)
South west	Batie	99.4 (98.3–99.9)	94.4 (89.9–97.4)	20.0 (12.7–28.9)	94.4 (85.4–99.3)	26.3 (06.3–54.5)	91.2 (85.3–95.7)	03.8 (01.6–07.3)

**Region**	**Health District**	**Diagnostic**			**Anti-Malaria Drugs**			
		Symptom	RDT	Microscopy	Oral ACT	Rectal or injectable	ITP-SP	ACT Out of Stock
	Dano	98.6 (97.3–99.4)	94.6 (91.5–96.7)	12.8 (09.1–17.3)	98.1 (94.9–99.7)	42.7 (21.8–65.5)	94.8 (92.1–97.0)	05.2 (03.2–08.0)
	Diebougou	99.2 (98.1–99.8)	94.8 (91.6–97.2)	15.9 (11.0–21.7)	96.8 (91.8–99.5)	22.0 (05.7–45.1)	93.3 (89.7–96.3)	04.2 (02.2–07.0)
	Gaoua	98.1 (96.6–99.2)	95.0 (92.1–97.3)	10.9 (07.3–15.3)	98.6 (96.0–99.8)	41.3 (21.8–62.5)	95.4 (92.7–97.5)	05.4 (03.1–08.4)
	Kampti	99.6 (98.6–100)	93.9 (87.9–97.6)	24.4 (14.6–36.5)	90.0 (72.2–99.1)	39.1 (08.6–78.2)	88.6 (79.4–94.9)	03.5 (01.2–07.6)

Table S3. Variables that best characterize each composite readiness profile.

	v.test	Mean		Standard deviation		*p*-Value
		In the profile	Overall	In the profile	Overall	
**Low Composite Readiness Profile**						
Diagnosis by microscopy	6.82	0.41	0.18	0.09	0.09	<0.001
ACT out of stock	2.89	0.06	0.05	0.03	0.01	0.004
Light source	2.11	0.79	0.66	0.08	0.17	0.035
Infant weighing scale	−2.52	0.63	0.76	0.10	0.14	0.012
Blood pressure apparatus	−2.79	0.97	0.98	0.02	0.01	0.005
Diagnosis by clinical symptoms	−3.34	0.96	0.98	0.04	0.02	0.001
Thermometer	−3.62	0.98	0.99	0.03	0.01	<0.001
Artesunate rectal or injectable forms	−4.15	0.18	0.39	0.06	0.14	<0.001
Rapid diagnostic test availability	−4.54	0.59	0.80	0.08	0.13	<0.001
Stethoscope	−6.37	0.95	0.98	0.03	0.01	<0.001
By rapid diagnostic test	−6.93	0.75	0.93	0.12	0.07	<0.001
Staff trained on malaria management	−7.52	0.75	0.95	0.09	0.07	<0.001
IPTp during pregnancy	−7.58	0.67	0.91	0.09	0.09	<0.001
Staff trained for IPT	−7.75	0.44	0.83	0.16	0.14	<0.001
First-line antimalarial (ACT)	−7.78	0.53	0.92	0.13	0.14	<0.001
**Medium Composite Readiness Profile**						
Rapid diagnostic test availability	−1.98	0.75	0.80	0.13	0.13	0.047
Diagnosis by microscopy	−2.02	0.14	0.18	0.04	0.09	0.043
Latex gloves for physical exam	−4.14	0.84	0.91	0.10	0.08	<0.001
Infant weighing scale	−4.31	0.64	0.76	0.12	0.14	<0.001
Light source	−5.80	0.47	0.66	0.15	0.17	<0.001
**High Composite Readiness Profile**						
Infant weighing scale	5.53	0.83	0.76	0.10	0.14	<0.001
Rapid diagnostic test availability	4.64	0.86	0.80	0.08	0.13	<0.001
Light source	4.03	0.73	0.66	0.11	0.17	<0.001
Latex gloves for physical exam	3.97	0.94	0.91	0.05	0.08	<0.001
Staff trained for IPTp	3.22	0.88	0.83	0.02	0.14	0.001
First-line antimalarial (ACT)	3.10	0.96	0.92	0.03	0.14	0.002
Staff trained on malaria management	3.02	0.97	0.95	0.01	0.07	0.003
IPTp during pregnancy	2.94	0.93	0.91	0.02	0.09	0.003
Diagnosis by Rapid diagnostic test	2.90	0.95	0.93	0.01	0.07	0.004
Stethoscope	2.56	0.99	0.98	0.00	0.01	0.010
Artesunate rectal or injectable forms	2.10	0.41	0.39	0.13	0.14	0.036
Diagnosis by microscopy	−2.37	0.16	0.18	0.05	0.09	0.018
